# Trichomoniasis as Seen in a Chronic Vaginitis Clinic

**DOI:** 10.1155/S1064744996000178

**Published:** 1996

**Authors:** Michael Dan, Jack D. Sobel

**Affiliations:** 1 Division of Infectious Diseases Department of Internal Medicine Wayne State University School of Medicine Detroit Medical Center Detroit MI USA; 2 Harper Professional Building Suite 2140 4160 John R Street Detroit MI 48201 USA

## Abstract

*Objective:* We sought to determine the clinical and laboratory features of trichomonas vaginitis (TV) in a chronic vaginitis clinic.

*Methods:* We studied 45 women with symptomatic TV attending a specialty chronic vaginitis clinic. These patients were older than the usual symptomatic patients with TV. They frequently described unusual chronicity of symptoms, half being referred because of clinical resistance and the other half referred because of chronic vaginitis of unknown etiology.

*Results:* In spite of the chronicity of infection, the signs and symptoms of florid inflammation were still evident and high numbers of polymorphonuclear leukocytes and parasitic load were present.

*Conclusions:* A longstanding infection, especially if previously untreated, invariably responded to conventional nitroimidazole therapy. In addition, the majority of patients seen with clinical resistance to the conventional doses of metronidazole responded to high-dose oral metronidazole therapy. Unsuspected TV should always be considered in low-risk patients with chronic vulvovaginal symptoms.


Infectious Diseases in Obstetrics and Gynecology 4:77–84 (1996)
© 1996 Wiley-Liss, Inc.

### Trichomoniasis as Seen in a Chronic Vaginitis Clinic

**Michael Dan and Jack D. Sobel**
*Division of Infectious Diseases, Department of Internal Medicine, Wayne State University School of Medicine, Detroit Medical Center, Detroit, MI*

### Abstract

*Objective:* We sought to determine the clinical and laboratory features of trichomonas vaginitis (TV) in a chronic vaginitis clinic.

*Methods:* We studied 45 women with symptomatic TV attending a specialty chronic vaginitis clinic. These patients were older than the usual symptomatic patients with TV. They frequently described unusual chronicity of symptoms, half being referred because of clinical resistance and the other half referred because of chronic vaginitis of unknown etiology.

*Results:* In spite of the chronicity of infection, the signs and symptoms of florid inflammation were still evident and high numbers of polymorphonuclear leukocytes and parasitic load were present.

*Conclusions:* A longstanding infection, especially if previously untreated, invariably responded to conventional nitroimidazole therapy. In addition, the majority of patients seen with clinical resistance to the conventional doses of metronidazole responded to high-dose oral metronidazole therapy. Unsuspected TV should always be considered in low-risk patients with chronic vulvovaginal symptoms.
© 1996 Wiley-Liss, Inc.

Key words
*Trichomonas vaginalis*, metronidazole, sexually transmitted disease, vulvovaginitis, parasites

Trichomoniasis (TV) is transmitted almost exclusively by sexual contact, representing a major health problem, especially for women.[1] Since the first description of TV by Donne in 1836,[3] its clinical features have been amply studied and critically reviewed.[3–6] With TV being a venereal infection, most studies examining its clinical manifestations have been carried out in sexually transmitted disease (STD) clinics.[4–6] STD clinics serve a selected population characterized by low socioeconomic status and a heavy burden of associated medical morbidity. Much less is known about the presentation of TV in other clinical settings. The findings reported here encompass the clinical and laboratory features of TV in a chronic vaginitis clinic.

### MATERIALS AND METHODS

The Wayne State University Vaginitis Clinic is a predominantly physician-referred service with approximately 1,000 patient visits per year. The clinical records were reviewed for patients with TV diagnosed by microscopy or culture from January 1990 through May 1994. Detailed medical histories were obtained from all patients, followed by general and pelvic examinations. After a careful vaginal and cervical examination using a speculum was carried out, vaginal swab specimens were obtained from both fornices and the mid-third of the vagina for a measurement of pH and saline and 10% potassium-hydroxide microscopic examination. Additional vaginal fluid samples were obtained for yeast and

Address correspondence/reprint requests to Dr. Jack D. Sobel, Harper Professional Building, Suite 2140, 4160 John R Street, Detroit, MI 48201.

Received February 9, 1996
Accepted July 8, 1996

*Trichomonas vaginalis* culture on Sabouraud's and Diamond's media, respectively. Cervical samples for *Neisseria gonorrhoeae* and *Chlamydia trachomatis* cultures were obtained if these infections were judged clinically relevant.

### Case Definition

Acute TV was defined as the presence of symptoms and signs compatible with vulvovaginitis ≤28 days and by the documentation of trichomonads on saline, wet-mount microscopy, or culture. Other diagnostic criteria included an increase in polymorphonuclear leukocytes (PMNs) in the vaginal secretions as detected by light microscopy (PMNs to squamous epithelial cells in a ratio of >1:1) and a vaginal pH of >4.5. Chronic TV included the same diagnostic criteria except that the duration of symptoms exceeded 1 month. A mixed infection; e.g., TV and candidiasis, was diagnosed if *Candida* organisms were visualized on microscopy and confirmed by culture in addition to the presence of *Trichomonas*. Concomitant bacterial vaginosis required the additional presence of clue cells on microscopy.

All male partners of the patients with both acute and chronic TV were given 2.0-g single-dose courses of metronidazole, regardless of previous anti-protozoal therapy.

### RESULTS

Forty-eight episodes of TV were diagnosed in 45 women at the Wayne State University Vaginitis Clinic during the study period. Three patients had 2 episodes of TV each. During the same period, 1,741 patients were seen at the vaginitis clinic. The 45 women with TV accounted for 2.5% of all patients seen and 4% of patients in whom a definite pathologic diagnosis was established. The most common diagnoses in the clinic were vulvovaginal candidiasis, found in 16% of the women, and bacterial vaginosis, observed in 12% of the patients (unpublished observations).

TV was diagnosed on the initial visit to the clinic in 29 of the 45 (64.4%) identified patients. Fifteen of these patients were referred with the diagnosis of TV unresponsive to conventional antitrichomonal therapy. The remaining 14 women in this group were referred because of undiagnosed persistent vaginal symptoms. In 16 additional patients (totaling 19 episodes), acute TV was diagnosed while the patient was being followed at the clinic for other categories of vulvovaginitis.

A mixed infection, namely, concomitant TV and bacterial vaginosis or TV and vulvovaginal candidiasis, was observed in 14 (34%) patients or 15 (31%) episodes. In 11 patients (73%) with mixed episodes, TV was associated with bacterial vaginosis; in 3 (20%) episodes, the concomitant infections included bacterial vaginosis and vulvovaginal candidiasis; and in 1 (7%) episode, vulvovaginal candidiasis was diagnosed concurrently. In an additional 3 episodes, yeasts were noted on culture but were not seen on the microscopic examination. Seven (47%) mixed infections were diagnosed on the first visit to the clinic (representing 24% of infections diagnosed on that occasion), and the remaining 8 episodes were observed on subsequent visits (42% of infections detected in returning patients). Most of the uncomplicated TV (21 of 30 or 70%) were diagnosed on the first visit.

The demographic data of the population studied are shown in Table 1. The mean age was similar in both TV subgroups. More than three-quarters of the patients with TV were black. Only a minority of the TV patients were married at the time of diagnosis.

Eighty-nine percent of the TV patients used some form of contraception; the single, most preferred device was the condom (28%), followed by the contraceptive pill (26%). About one-third of the women with TV relied on sterilization (hysterectomy, tubal ligation, menopause, or vasectomy) for contraception. Of the women with TV, 92.5% had had at least 1 sexual partner in the year preceding the diagnosis. The frequency of sexual activity in the TV group was variable: 54% of the patients had coitus <once per week, while 21.6% had 2 encounters per week and 19% had intercourse >3 times per week. A low frequency (<once per week) of intercourse was reported more often by women diagnosed on initial visits than in patients diagnosed on subsequent visits (74% vs. 25%).

A history of TV was obtained in 19 (42%) patients, including 3 patients in whom previous episodes of TV were diagnosed at the clinic. Other past genital infections were frequent: 35% reported past candidiasis or bacterial vaginosis; 11% had had an STD; and 25% had had both.

The prevalence of vaginitis-associated symptoms in the study population is shown in Table

**TABLE 1. Demographic data on patients with TV**

| | All patients (N = 45) | Patients diagnosed on first visit (N = 29) "chronic" | Patients diagnosed on subsequent visits (N = 16) "acute" |
|---|---|---|---|
| Mean age (range) (years) | 36 (18–68) | 34.9 (18–68) | 36.1 (21–52) |
| White/black (% black) | 9/34 (79%) | 6/21 (78%) | 3/13 (85%) |
| Married at diagnosis (%) | 8/45 (18%) | 3/29 (10%) | 5/16 (26%) |
| Unemployed (%) | 4/39 (10%) | 3/28 (11%) | 1/11 (9%) |

**TABLE 2. Frequency of symptoms in patients with TV**

| | All episodes (N = 48) | Episodes diagnosed on first visit (N = 29) "chronic" | Episodes diagnosed on subsequent visits (N = 19) "acute" |
|---|---|---|---|
| Duration | | | |
| ≤4 weeks | 16/43 (37%) | 1/27 (4%) | 15/16 (93%) |
| ≥4 weeks | 27/43 (63%) | 26/27 (96%) | 1/16 (7%) |
| Odor | 32/42 (81%) | 18/23 (78%) | 16/19 (84%) |
| Itching | 36/45 (80%) | 20/27 (74%) | 16/18 (89%) |
| Irritation | 7/36 (19%) | — | — |
| Burning | 15/41 (37%) | — | — |
| Dyspareunia | 8/20 (40%) | — | — |

2. Since women attend vaginitis clinics primarily because of symptoms, all our patients were symptomatic. Most women (63%) had prolonged or chronic symptoms (>4 weeks). Chronic symptoms were particularly evident in patients diagnosed with TV on the first visit to the clinic (96%), while only 7% of the episodes observed on subsequent visits to the clinic were chronic. Eight patients, all with uncomplicated TV and chronic symptoms, had not had sexual contact for more than 6 months (4 patients for ≥1 year and 1 patient for 3 years). In the chronic cases, the prolonged duration of symptoms was caused by the lack of an appropriate diagnosis and, hence, therapy (14 episodes) or by a nonresponse to the conventional treatment (15 episodes).

Malodor and itching, the most common symptoms, were reported as often in chronic as in acute infections, even after the exclusion of mixed episodes (78% and 84%, respectively, for odor; 74% and 89%, respectively, for itching). Irritation, burning, and dyspareunia, which were less frequently described, were not further analyzed because of the small numbers. The prevalence of the physical findings in our patients with TV is described in Table 3. An abnormal vaginal discharge was observed in all episodes. Most patients had mild discharges, with no difference observed between chronic and acute infections, even after the exclusion of mixed infections. The color of the discharge was yellow or yellow-green in approximately half of the episodes, while, in the remaining cases, it was white. A yellow discharge was as common in chronic (55%) as in acute episodes (50%). The discharge was described as frothy in only 4 instances and cheese-like in another 2 patients. The signs of vulvar inflammation (erythema ± edema), which were observed in 70% of the episodes, were somewhat more common in chronic than in acute infections, even after the elimination of mixed cases (77% vs. 59%). Vaginal erythema (±edema) was noted in 90% of episodes; all 5 cases with intense inflammation (erythema + edema) had chronic courses. Cervical involvement was less common (22%), and colpitis macularis was observed in a single case with an acute presentation (3%).

The laboratory findings associated with TV are presented in Table 4. An elevated pH (>4.5) was measured in all but 1 case, while a positive amine test was detected in three-quarters of the episodes. Leukocyte increase and abnormal bacterial flora

TABLE 3. Frequency of various signs in patients with TV

| | All episodes (N = 48) | Episodes diagnosed on first visit (N = 29) "chronic" | Episodes diagnosed on subsequent visits (N = 19) "acute" |
|---|---|---|---|
| Discharge | | | |
| + | 31/48 (64%) | 18/28 (64%) | 12/19 (63%) |
| ++ | 5/48 (10%) | 4/28 (14%) | 1/19 (5%) |
| +++ | 11/48 (24%) | 5/28 (18%) | 6/19 (32%) |
| Color | | | |
| Yellow-green | 19/36 (53%) | 12/22 (55%) | 7/14 (50%) |
| Vulva | | | |
| Normal | 13/43 (30%) | 6/26 (23%) | 7/17 (41%) |
| Erythema | 19/43 (44%) | 12/26 (46%) | 7/17 (41%) |
| + Edema | 11/43 (26%) | 8/26 (31%) | 3/17 (18%) |
| Vagina | | | |
| Normal | 4/41 (10%) | | |
| Erythema | 32/41 (78%) | | |
| + Edema | 5/41 (12%) | | |
| Cervix | | | |
| Normal | 25/32 (78%) | | |
| Erythema | 7/32 (22%) | | |

(loss of the dominant lactobacillus morphotype) were observed on microscopic examination in the majority of infections. In only 2 instances was *T. vaginalis* detected by culture after a negative wet-mount examination. It appears that, with the exception of the amine test, the laboratory findings were as pronounced in chronic as in acute infections (after the exclusion of mixed infections): a pH of >5 was observed in 83% of the chronic episodes vs. 69% of the acute ones; increased numbers of leukocytes and *T. vaginalis* organisms were noted as often in chronic protracted TV as in acute TV.

Four different regimens of metronidazole were used in the treatment of TV: 2.0-g single-dose (1 case); 7-day regimen of metronidazole, 500 mg b.i.d. (28 cases); and 14-day regimen with (9 cases) or without (2 cases) vaginal metronidazole. In 8 episodes, there was insufficient information on the dosing modality present in the chart. Most of the long courses (14 days) were prescribed for patients referred because of a history of failure of the standard regimens. The only patient with an acute presentation, who received a prolonged course of oral metronidazole, had been successfully treated at the clinic 2 years earlier for TV with a laboratory-proven resistant organism and failed to respond to a standard regimen given elsewhere for the present episode (see illustrative case 1). The 7-day metronidazole regimen was equally used for chronic and acute episodes. Two patients successfully responded to high-dose (>2.0 g/day 7-day regimens). Both of these patients had been referred to the clinic because of failure to respond to multiple courses of metronidazole. Two patients had particularly stubborn infections. In 1 case (illustrative case 2), no response was noted after several courses of metronidazole, while a course of tinidazole was immediately successful. In the other case (illustrative case 3), the organism demonstrated in vitro resistance to metronidazole. This patient failed to respond to several courses of the drug given over 18 months, until she was lost to follow-up.

### ILLUSTRATIVE CASES

### Case 1

A 21-year-old single woman was first seen in the clinic in December 1990 after having been treated by several physicians over a period of 12 months for resistant TV. Her complaints consisted of malodorous discharge, itching, and burning. She had abstained from sexual intercourse since her symptoms began. The patient's other medical problem was epilepsy for which she had been taking phenobarbital, phenytoin, and sodium valproate. The vulva, vagina, and cervix appeared intensely erythematous, and a yellow-green discharge was noted. The pH of the vaginal fluid was 6. On wet-mount examination, an abundance of PMNs and trichomo-

TABLE 4. Frequency of laboratory findings in patients with TV

| | All episodes (N = 48) | Episodes diagnosed on first visit (N = 29) "chronic" | Episodes diagnosed on subsequent visits (N = 19) "acute" |
|---|---|---|---|
| pH | | | |
| <4.5 | 1/45 (2%) | 1/29 (3%) | 0 |
| 4.5–5 | 9/45 (20%) | 4/29 (14%) | 5/16 (31%) |
| >5 | 35/45 (78%) | 24/29 (83%) | 11/16 (69%) |
| Amine test | | | |
| Positive | 30/40 (75%) | 17/25 (68%) | 12/15 (80%) |
| WBCs[a] | | | |
| − | 6/40 (15%) | 2/29 (7%) | 4/11 (36%) |
| + | 34/40 (85%) | 27/29 (93%) | 7/11 (64%) |
| Bacterial flora[b] | | | |
| Normal | 6/33 (18%) | 6/24 (25%) | 0/9 (0%) |
| Abnormal | 24/33 (73%) | 16/24 (67%) | 8/9 (89%) |
| Mixed | 3/33 (9%) | 2/24 (8%) | 1/9 (11%) |
| Trichomonads[c] | 46/48 (96%) | 27/29 (93%) | 19/19 (100%) |

[a]Saline microscopy – ratio of PMNs: epithelial cell >1.0.
[b]As determined on Gram's stain.
[c]Visualized on saline microscopy.

nads were observed, while no clue cells or yeasts were present. Metronidazole, 250 mg t.i.d., was prescribed for 10 days. The patient improved while on therapy, but her symptoms recurred 2 weeks later when trichomonads were again seen on the wet-mount examination. Metronidazole susceptibility testing of the initial isolate revealed a minimum lethal concentration of 80 μg/ml. Two subsequent courses of high-dose oral metronidazole (500 mg b.i.d. for 14 days together with 500 mg b.i.d. vaginally for 7 days) also failed to eradicate the infection. The infection was finally cured by the oral administration of metronidazole, 500 mg q.i.d., over 2 weeks, accompanied by local vaginal metronidazole therapy. She was subsequently seen at 3 and 6 months with no evidence of symptomatic or parasitic recurrence.

*Comment:* The failure to eradicate an infection with the standard dose of oral metronidazole can be attributed to the relative in vitro resistance of the parasite. However, another factor that might have been contributory was the patient's phenobarbital therapy, as the drug is reported to alter metronidazole hepatic metabolism (see Discussion).

### Case 2

A 40-year-old married woman presented with a 3-month history of purulent discharge, itching, and soreness that was diagnosed as TV. She had previously failed to respond to 2 courses of metronidazole, 500 mg q.i.d. for 14 days. On her examination, intense vulvovaginal inflammation and an abnormal discharge were noted. The vaginal pH was >6 and the amine test was negative. Numerous PMNs and trichomonads were observed on the wet-mount examination. The patient was treated with metronidazole orally, 1.0 g q.i.d., and vaginal tablets, 500 mg b.i.d. for 14 days (a total dose of 70 g). One week after the completion of treatment, her symptoms recurred. Parasites were again seen on the microscopic examination. A hospital admission for intravenous (IV) metronidazole therapy was refused by the patient. Finally, tinidazole, 2.0 g orally for 14 days, was prescribed. Her response to tinidazole was dramatic and her follow-up examinations were normal at 1 and 3 months.

*Comment:* In this case, TV persisted despite multiple courses of metronidazole, including a combination of high-dose oral and intravaginal administration. In vitro susceptibility studies were not performed. A prompt response was observed to tinidazole, another 5-nitroimidazole.

### Case 3

A 24-year-old single woman was referred to the clinic for recurrent TV that had been present for 1 year. She had received at least 6 courses of metronidazole, 500 mg b.i.d. for 7–14 days. The patient had not been sexually active during the entire period because of dyspareunia. When seen at the clinic, she complained of a green-yellow discharge, malodor, burning, and itching. On her examination, the vulva and vagina were erythematous and edematous. Abnormal secretions were observed, with a pH of >6 and a negative amine test. The wet-mount examination revealed an abundance of PMNs and trichomonads. The patient was started on metronidazole tablets, 3.0 g/day orally and 1.0 g/day by the intravaginal route. Because of severe nausea, the dose of the oral drug was reduced to 2.0 g/day. After completing a 14-day course, her partner being treated concomitantly, the clinical and laboratory findings persisted. The patient was lost to follow-up and then hospitalized 4 months later, at which time she continued to have a florid TV. A total of 26.0 g of metronidazole was administered IV over 7 days with no effect on the TV. The minimal inhibitory concentration (MIC) of the trichomonal isolate was 265 μg/ml. The patient was last seen at the clinic in September 1990 and lost to follow-up.

*Comment:* The infection in this case was particularly resistant to modalities of metronidazole therapy, including high-dose IV administration. At the time, tinidazole therapy was not available.

### DISCUSSION

The majority of studies that have examined the demographic, clinical, and laboratory aspects of TV were conducted at STD clinics.[4–6] It is not surprising, therefore, that some of the findings in the present investigation, which was conducted in a referral chronic vaginitis clinic, differ from those previously reported.

The mean age of the patients treated at our clinic (36 years) was higher than that of women diagnosed at STD clinics (24 years).[4–6] Most of our patients were employed, which is probably related to the socioeconomic level of the population attending our clinic, with most of the patients belonging to the middle class. In keeping with previous studies performed in STD clinics in the United States, there was a high proportion of unmarried patients and a predominance of black women.[4–6] These findings are in sharp contrast with the characteristics of the patients with vulvovaginal candidiasis seen on our referral clinic who are mostly married and white.[(7)] Noteworthy, there was a common association of TV with bacterial vaginosis rather than vulvovaginal candidiasis. Fouts and Kraus[4] have also noted that vaginal yeast is detected less frequently with TV. Finally, in contrast to previous studies of TV in STD clinics, a higher proportion of our patients used barrier contraception (28%) compared with previous reports (<6%).[5,6]

Although sporadic cases of persistent TV have been reported,[8–10] no attempt has been made to study the clinical and laboratory characteristics of TV in its more chronic form. In the present study, chronic TV is best represented by a group of 8 women with prolonged symptoms for at least 6 months (6–36 months). During the symptomatic period, the patients were not sexually active; accordingly, reinfection can be ruled out. The prolonged reduction of sexual activity or abstinence in patients with chronic diseases deserves special attention. In this regard, it should be noted that dyspareunia was as common in the group of patients with chronic disease as in those with acute symptoms. No significant difference was observed between the patients with chronic presentations and those with acute diseases with regard to the symptoms and signs with the exception of a tendency toward more intense vaginal inflammation in some chronic cases. The most common symptoms among all our patients were malodorous discharge and itching, each reported by 80% of the women with either acute or chronic presentation. This prevalence is somewhat higher than reported by others.[6] Similarly, vulvar and vaginal erythema was more often observed in our patients than in those reported previously.[4,6]

No difference in laboratory findings was evident in the patients with acute or chronic TV. Thus, in spite of chronicity, no loss in infectious load or reduction in inflammatory reaction was apparent.

Metronidazole has been the drug of choice for treating trichomonal infections since its introduction in the 1960s. Therapeutic regimens using oral metronidazole, 250 mg t.i.d., 500 mg b.i.d. for 7 days, or, more recently, a single 2-g dose, have shown cure rates of >90%.[1] Based on our limited experience, it appears that the standard 7-day metronidazole regimen is efficacious in chronic, untreated vaginal TV.

Although TV can be refractory to treatment with metronidazole, true resistance of the organisms to metronidazole has remained uncommon. Failure to eradicate *T. vaginalis* has been attributed to poor patient compliance, reinfection by an untreated or new partner, interference by other organisms present on the vagina,[11,12] possible drug interaction,[13] or low serum zinc concentrations.[14] A repeat course of a conventional dose, 7-day regimen or a longer (14-day) course, together with a correction of the failure-predisposing causes if relevant, should cure the vast majority of patients with recurrent TV.

True laboratory-documented metronidazole-resistant *T. vaginalis* isolates were first described in the late 1970s and early 1980s. The mechanism of resistance appears to be related to a deficiency in the ferredoxin-linked, oxygen-scavenging capacity which limits the activation of metronidazole by reducing its nitro group.[15] In vitro studies have shown that mebendazole, furazolidone, and ansomycin are the most active drugs against metronidazole-resistant trichomonads.[16] Clinical resistance, is initially treated with an increase in the daily dose (including IV administration) and duration of treatment. This approach may be hampered, however, by side and toxic effects, the frequency and severity of which are dose-dependent.[10] Although not tested in prospective studies, patients with clinical resistance also appear to benefit from the concomitant intravaginal administration of metronidazole.[8] Alternative methods with variable success include systemic tinidazole,[10] mebendazole,[10] 20% saline douches, 3% acetic-acid douches, intravaginal application of gentian violet, chlorhexidine, povidone-iodine, hydrogen peroxide, nonoxynol-9, nitrofurantoin, and clotrimazole.[9]

One of our refractory cases responded promptly to tinidazole after the failure of several courses of metronidazole (in vitro susceptibility was not tested), while another patient with true drug resistance remained unresponsive to multiple high-dose regimens of metronidazole until she was lost to follow-up. A true resistance to metronidazole is, fortunately, rare, and managing patients infected with such strains can be very frustrating. The large choice of therapeutic substitutes with limited efficacy clearly indicates the need for further research to find an effective alternative treatment.

Clinicians in a referral chronic vaginitis clinic might be expected to see infrequent cases of clinically resistant TV but should also be aware of the possibility of undiagnosed, missed cases of susceptible TV. This report serves to emphasize the frequent chronic nature of this infection when undiagnosed, particularly in older, low-risk women, resulting in considerable morbidity. The failure to diagnose TV reflected by the substandard use of available diagnostic methods is regrettable, given the rapid response of chronic vaginitis to conventional therapy.

### REFERENCES

1. Rein MF, Muller M: *Trichomonas vaginalis* and trichomoniasis. In Holmes KK, Mardh P-A, Sparling PF, Weisner PJ (eds): Sexually Transmitted Diseases. 2nd ed. New York: McGraw-Hill International Book Co., pp 481–492, 1990.
2. Wisdom AR, Dunlop EMC: Trichomoniasis: Study of the disease and its treatment in women and men. Br J Vener Dis 41:90–96, 1965.
3. Donne MA: Animalcules observés dans les matières purolentes et le produit des secfetions des organes genitaux de l'homme et de la femme. CR Acad Sci 3:385–386, 1836.
4. Fouts AC, Kraus SJ: *Trichomonas vaginalis:* Re-evaluation of its clinical presentation and laboratory diagnosis. J Infect Dis 141:137–143, 1980.
5. Spence MR, Hollander DH, Smith J, McCaig L, Sewell D, Brockman M: The clinical and laboratory diagnosis of *Trichomonas vaginalis* infection. Sex Trans Dis 7:168–171, 1980.
6. Wolner-Hansen P, Krieger JN, Stevens CE, et al.: Clinical manifestations of vaginal trichomoniasis. JAMA 261:571–576, 1989.
7. Geiger AM, Foxman B, Sobel JD: Chronic vulvovaginal candidiasis: Characteristics of women with *Candida albicans, Candida glabrata* and no *Candida*. Genitourin Med 71:304–307, 1995.
8. Grossman JH, Galask RP: Persistent vaginitis caused by metronidazole-resistant trichomoniasis. Obstet Gynecol 76:521–522, 1990.
9. Livengood CH, Lossick JG: Resolution of resistant vaginal trichomoniasis associated with the use of intravaginal nonoxynol-9. Obstet Gynecol 78:954–956, 1991.
10. Hamed KA, Studemeister AE: Successful response of metronidazole-resistant trichomonal vaginitis to tinidazole. Sex Trans Dis 19:339–340, 1992.
11. Ahmed-Jushuf IH, Murray AE, McKoewn J: Managing trichomonal vaginitis refractory to conventional treatment with metronidazole. Genitourin Med 64:25–29, 1988.
12. Edwards DI, Thompson EJ, Tomusagne J, Shanson D: Inactivation of metronidazole by aerobic organisms. J Antimicrob Chemother 5:315–316, 1979.
13. Mead PB, Gibson M, Schentag JJ, Ziemniak JA: Possible alteration of metronidazole metabolism by phenobarbital. N Engl J Med 306:1490, 1982.
14. Wilmott F, Say J, Downey D, Hookam A: Zinc and recalcitrant trichomoniasis. Lancet 1:1053, 1983.
15. Yarlett N, Yarlett NC, Lloyd D: Ferredoxin-dependent reduction of nitromidazole derivatives in drug-resistant and susceptible strains of *Trichomonas vaginalis*. Biochem Pharmacol 35:1703–1708, 1986.

16. Sears SD, O'Hare J: In vitro susceptibility of *Trichomonas vaginalis* to 50 antimicrobial agents. Antimicrob Agents Chemother 32:144–146, 1988.
